# To live with a wagging tailed assistant – Service and hearing dog ownership from the perspective of Swedish owners

**DOI:** 10.1111/hsc.13128

**Published:** 2020-08-11

**Authors:** Martina Lundqvist, Lars‐Åke Levin, Jenny Alwin, Ann‐Charlotte Nedlund

**Affiliations:** ^1^ Department of Health, Medicine and Caring Sciences Unit of Health Care Analysis Linkoping University Linköping Sweden

**Keywords:** assistance dog, assistive technology, health technology assessment, qualitative analysis, thematic content analyses

## Abstract

Individuals who have a functional or health impairment, are often in great need of conventional healthcare, social care and support, as well as help from family and friends. The use of dogs may be an important assistive support for this population. Assistance dogs are trained to assist with their owners’ specific needs. The aim of this study is to explore service and hearing dog ownership from the owner´s perspective, by examining the owner's expectations before training a dog, and experiences after having a certified dog. This study was designed as a longitudinal intervention study with pre‐post design. The participants included in the study trained their own dogs to become service or hearing dogs. A number of open‐ended questions were answered by the participants before the training of the dog started, three months after the dog was certified, and 1–3 years after the first follow‐up. Data were analysed using thematic content analysis. The participants’ expectations of what the dog would contribute after being certified was high. Their perceived experiences in many ways reflected their expectations. For example, they perceived that the dog contributed to improved health status, a more active lifestyle, improved ability to feel secure, and that they had become more independent. They also felt that the dog had strengthened their social relationships. Negative experiences were also identified. Not being allowed to bring their dog into public places and negative attitudes from other people were examples of this. This study shows that individuals being supported by an assistance dog experience the dog as an invaluable help in their everyday life. By improving the owners’ lives in many ways, a certified service or hearing dog is a novel and important assistive support for people with a functional or health impairment.

**What is known about this topic**

- Assistance dogs may have a positive impact on health related quality of life, well‐being and activity level
- Assistance dogs improve self‐esteem and have a positive impact on an individual's psychosocial functioning
- Identified drawbacks with assistance dog ownership are public access, lifestyle adjustments and dog behaviour

**What this paper adds**

- The assistance dog ownership affects the participants’ perceived health status in a positive way
- The assistance dog enables the participants to feel safer and more independent in their everyday life
- The assistance dog contributes to negative experiences such as restrictions on bringing the dog into public places and negative attitudes from other people

## 1 INTRODUCTION

This article focuses on individuals with a functional or health impairment who receive an assistance dog. Having a functional or health impairment often means being in great need of conventional healthcare, social care and support, as well as being in need of help from family and friends (McPhail, 2016). Having a disability also increases the risk of social isolation and thereby a more restricted lifestyle (Tough, Siegrist, & Fekete, 2017).

An assistance dog as an assistive support can be used by people with different diseases and/or functional impairments since the training of the dog, and thus what they can help with, can be customised to the needs of the owner. The assistance dogs term includes three types of dogs: guide dogs, hearing dogs and service dogs. Furthermore, the service dog concept can be divided into subcategories such as physical service dogs, diabetes alert dogs, seizure alert dogs etc. (Assistance Dogs International, 2018). Physical service dogs assist people with functional impairments, for instance they help with getting dressed, pick up dropped items, provide balance and stability. Moreover, they are also commonly trained to attract another person's attention in case of emergency or if the owner needs help. The diabetes alert dogs and the seizure alert dogs are trained to warn their owner of high and low blood sugar levels and of imminent epileptic seizures, respectively. A hearing dog can assist people who have hearing impairments or who are deaf. They are trained to alert their owner of important sounds such as smoke alarms, a ringing telephone, doorbells etc. (The Swedish Association of Service Dogs, 2017; The Swedish Kennel Club, 2017) In this study, the focus will be on service and hearing dogs in a Swedish context.

In Sweden, there are three possible ways to obtain a certified service or hearing dog. The most common is that the owner in collaboration with a certified instructor trains a dog (The Swedish Working Dog Association (personal communication, June 23, 2020)). In between the training sessions with the instructor, the owner must train the dog on her own. A second possible way is to purchase a dog that has been fully trained by a certified instructor. If this is the case a match between the dog and the intended owner is made based on a number of criteria and when the dog is judged to be ready to meet the new owner, the instructor trains the dog and the intended owner together for a while (The Swedish Working Dog Association, 2020a, 2020b). Finally, the owner can conduct the training of a dog on their own. All situations require that the owner, together with the dog, passes an examination. Only then it is possible to get the vest as proof that the dog is certified. To keep the dog certified the dog and the owner must pass an annual certification maintenance test (The Swedish Working Dog Association, 2020a, 2020b). The test is conducted to ensure that the dog and the owner maintain the standards. The Swedish Agency for Participation is responsible for financing and controlling a support function that can guarantee the quality/standard of the assistance dogs and their training (The Swedish Agency for Participation, 2020). Today that support function is the Swedish Working Dog Association.

Sweden has three levels of government: national, regional and local. At the regional level there are 21 regions and at the local level there are 290 municipalities. Both regions and municipalities have their own self‐governing local authorities which are responsible for different activities in the community (Swedish Association of Local Authorities & Regions, 2020). In Sweden today, there are no national regulation regarding how to finance assistance dogs as supporting aids. In addition, guidelines on how regions or municipalities can contribute with financial support is lacking (1177 Vårdguiden, 2020). Funding of a service or hearing dog is therefore mostly achieved by grants, or by the owner personally. However, the regions and municipalities do have the responsibility to provide individuals, with functional or health impairment, individually adapted care and rehabilitation. Hence, it is possible to give financial support, both in terms of grants for the purchase cost or grants for the training cost (The Swedish Working Dog Association, 2020a, 2020b). Another aspect not nationally regulated is the assistance dog public access rights, this is instead decided by the local municipalities (The Swedish Kennel Club, 2020). Access to public places can therefore differ depending on where in Sweden you are. Furthermore, access to restaurants and grocery shops is recommended to be permitted based on existing guidelines from the National Food Agency (The National Food Agency, 2006). These guidelines state that assistance dogs should be allowed as long as it does not pose a risk for food to become contaminated. However, since it is not regulated in Swedish law, the shopkeeper can deny access.

Findings from previous research have shown that the use of dogs may be an important assistive support for this population (Lundqvist, Levin, Roback, & Alwin, 2018). It has been shown that assistance dogs may have a positive impact on health related quality of life (HRQoL), well‐being and activity level (Hall, MacMichael, Turner, & Mills, 2017; Lundqvist et al., 2018; Shintani et al., 2010). In addition, it has been suggested that assistance dogs improve self‐esteem and have an impact on an individual's psychosocial functioning (Allen & Blascovich, 1996; Collins et al., 2006). A number of studies have used qualitative methods to explore the use of assistance dogs (Camp, 2001; Herlache‐Pretzer et al., 2017; Rodriguez, Bibbo, Verdon, & O’Haire, 2019). In summary, the studies identified both benefits and drawbacks with having an assistance dog. As a complement to previous research, this study will use qualitative data to explore in what way assistance dog enable improvements found in HRQoL, well‐being and activity level. The meaning of assistance dog ownership from the owner's perspective is an important aspect to understand in‐depth the use of an assistive dog as a supportive aid.

The aim of the present study is to explore service and hearing dog ownership from the owner´s perspective by examining the owner's expectations before training a dog, and experiences after having a certified dog.

## 2 METHODS

This study is based on the Service and Hearing Dog Project, which has been reported previously (Lundqvist et al., 2018). The intention of the project was to give the participants, in collaboration with a certified instructor, the possibility to train a companion dog to become a certified service or hearing dog.

The Service and Hearing Dog Project was a longitudinal intervention study with pre‐post design. The inclusion criteria for participating in the study were (a) ≥16 years old, (b) having a companion dog and (c) needing a service or hearing dog. People interested in participating in the study reported their interest to the Swedish Association of Service Dogs (SAF), i.e. the sample was self‐selected. To determine the dog's appropriateness and to evaluate whether the participant would be able to carry out the training of the dog, a minor suitability test was conducted supervised by SAF. If the minor test was passed, SAF mediated the contact between the participant and the research group. At that point, the participant gave written informed consent to participate in the study. The recruitment of participants started in 2009 and lasted until 2013. In total fifty‐five participants were included in the study. The participants had an average age of 44 years when entering the study. The majority were females. The most common reasons for needing a certified dog were diabetes, neurological disorders and musculoskeletal disorders, table 1. The participants included in the study had a poor perceived health‐related quality of life (HRQoL) (Lundqvist et al., 2018). Comparing the participants HRQoL scores with HRQoL scores for the general population showed that the participants had scores that were remarkably low (Lundqvist et al., 2018). The poor HRQoL indicated that the participants were in bad condition with substantial needs.

**Table 1:** Characteristics of the participants and the dogs

Baseline characteristics, participants Total (*n* = 55)		Baseline characteristics, dogs Total (*n* = 55)	
Age (years)		Age (years)	
Mean	43.8	Mean	2.2
Min – max	17 – 68	Min‐max	1.3 – 4.0
Gender		Gender	
Female	85.5%	Bitch	49.1%
Education		Weight (kg)	
Primary school	10.9%	Mean	22.6
Secondary school	27.3%	Min – max	3,2 – 52.0
Post‐secondary education	21.8%	Height (cm)	
University degree	40.0%	Mean	49.8
Main activity/professional status		Min – max	19.0 – 67.5
Employed full‐time	9.1%	Assistance dog type	
Employed part‐time	23.6%	Physical service dog	54.5%
Student	7.3%	Diabetes alert dog	36.4%
Sick leave	7.3%	Seizure alert dog	3.6%
Retired	3.6%	Hearing dog	5.5%
Disability pension	41.8%	Breed categories^a^	
Other	7.3%	Retrievers ‐ Flushing Dogs ‐ Water Dogs	38.2%
Household arrangement		Sheepdogs and Cattle Dogs	21.8%
Household of more than 1	60.0%	Companion and Toy Dogs	16.4%
Single	40.0%	Pinscher and Schnauzer ‐ Molossoid Breeds ‐ Swiss Mountain and Cattle Dogs	7.3%
Disease/functional impairment		Terriers	7.3%
Diabetes	36.4%	Sighthounds	3.6%
Neurological disorder	27.3%	Crossbreed	3.6%
Musculoskeletal disorder	21.8%	Spitz and Primitive types	1.8%
Deaf/hard of hearing	5.5%		
Epilepsy	3.6%		
Other	5.4%		

^a^ According to Federation Cynologique Internationale (FCI) (Federation Cynologique Internationale, 2018).

The dogs included in the study are presented in table 1. Fifty‐five percent were trained as physical service dogs, thirty‐six percent as diabetes alert dogs, four percent as seizure alert dogs and six percent as hearing dogs. The two most common breed categories in the study were ‘Retrievers – Flushing Dogs – Water Dogs’ and ‘Sheepdogs and Cattle Dogs’.

Prior to the start of the training of the dog, the participant and the dog also undertook a major suitability test. It determined the dog's responsiveness and obedience. At that time (baseline), the participants received a postal questionnaire that included two open‐ended questions. These open‐ended questions concerned the participant's expectations of training a certified service or hearing dog, and their thoughts about how the dog would influence their situation after becoming certified [Additional file 1].

Within the project the training of the dog took on average 1.5 years before the certification test was taken. It included tests to assess that the dog had certain required skills. Three months after the dog became certified (follow‐up), the participants received an additional postal questionnaire. On this occasion, the participants answered six open‐ended questions. The questions were related to their experience of their certified dog in terms of social situation, well‐being, possibility to undertake activities, medical situation and negative experiences [Additional file 2]. Fifty‐three participants completed and returned their baseline and first follow‐up survey. Two participants did not answer the surveys.

A second long‐term follow‐up was conducted somewhere between 1–3 years after the first follow‐up. It was sent to all the intended participants simultaneously. Hence the difference in follow‐up time. The questionnaire was sent by post and contained the same open‐ended questions that were asked at the first follow‐up [Additional file 2]. Thirty‐three participants received the second follow‐up survey, of which 21 returned the questionnaires.

All participants who had not answered any of the questionnaires received one or two reminders after the initial questionnaire had been sent. The research group constructed the questions.

### 2.1 Analysis

A thematic content analysis of the open‐ended questions was carried out. The analysis was conducted in accordance with Braun and Clark (Braun & Clarke, 2006). An interlay inductive approach was used to identify patterns and categorise the quotes into themes. To reduce the risk of the analysis being driven by the questions, the answers were separated from the questions. In order to still be able to deduce the answer to a specific question, all answers were colour‐coded before the questions were removed. However, the colours were not considered when conducting the analysis. To familiarise and get a sense of the data the author who conducted the analysis (ML) read the answers several times. Thereafter, the sorting of the answers was conducted based on identified patterns perceived as important in relation to the overall research question. To ensure transparency and to minimise the risk for the analyse being influenced by ML’s perception, the analytic process was constantly discussed with A‐CN. Furthermore, the analysis was reviewed by the research group. Together they interpreted the data to check if ML had identified all potential themes and sub‐themes and how they fit together. The group also discussed the essence of the themes and helped with naming the themes. Using different analysts to interpret data is a form of triangulation that validates the analysis (Ritchie, Lewis, McNaughton, & Ormston, 2014).

Quotes were translated from Swedish to English. All quotes were anonymised. The participants are denoted with P if they have a physical service dog, with D if they have a diabetes alert dog, with S if they have a seizure alert dog and with H if they have a hearing dog. The letter indicating assistance dog type is followed by a unique number referring to a specific participant. If the designation is preceded by an L, it refers to an answer given at the long‐term follow‐up.

The study was approved by the regional ethics vetting board Linköping University (No: 157‐09) and retrospectively registered in clinicaltrial.gov, NCT03270592, September 2017.

## 3 FINDINGS

In the following section, we highlight the expectations that the participants had before training the dog. In addition, we present their perceived experiences, positive as well as negative, after the training of the dog was complete and the dog was certified as a service or hearing dog. The perceived experiences will be presented both in the short‐term (three months after the dog was certified) and in the long‐term (1–3 years after the first follow‐up). Table 2 summarise the findings.

**Table 2:** Expectations brought up before the training of the dog started and perceived positive and negative experiences brought up after the training of the dog was complete

Themes	Sub‐themes	Sub‐sub‐themes
Expectations	The skills of the dog	‐ Alarm for low and high blood sugar levels ‐ Assistance in everyday life ‐ Epileptic seizure
	Security and independence	‐ General security ‐ Specific security
	The training	‐ The importance of the instructor ‐ The training of the dog ‐ Support and help to train my dog
	Overall life changes	
Positive experiences at short‐term follow‐up	Health status	‐ Impact on general condition ‐ Prevents epileptic seizures ‐ Stable blood sugar levels ‐ Less anxiety ‐ Mobility ‐ I can save my physical resources, it gives me less pain
	Being active	‐ I´m more active, I have to because of the dog ‐ The dog has made it possible for me to be more active ‐ No difference
	Security and independence	‐ General security ‐ Specific security ‐ I don´t have to rely on others ‐ The environment has been enlarged
	Social relationships	‐ Positive ‐ No impact at all
	The dog is the best thing that has happened to me	‐ Life changing
Negative experiences at short‐term follow‐up	Negative attitudes	‐ Offensive comments
	Limited/denied access to public places	‐ We are not welcome everywhere
	Reduced freedom	‐ Self‐perceived obstacles
	Feeling stressed	‐ Someone else to take care of
	The training	
Positive experiences at long‐term follow‐up	Health status	‐ Decreased mental illness ‐ Stable blood sugar levels ‐ Reduced pain and reduced use of analgesics ‐ Fewer epileptic seizures ‐ Less drugs
	Being active	‐ My service dog makes my life so much more active ‐ Dog exercises ‐ I exercise
	Security and independence	
	Social relationships	‐ Positive ‐ No impact at all
	Support	‐ The dog makes it easier
	Care	‐ Someone to cherish
	The training	‐ Good trainers
Negative experiences at long‐term follow‐up	Negative attitudes	‐ Other people’s lack of knowledge about these dogs
	Limited access to public places	‐ I have had to leave shops
	Controlling the dog	‐ The dog is hard to control in certain situations
	Hygiene/dirty at home	

### 3.1 Expectations before

The expectations we identified were categorised into sub‐themes, which consistently reflected the participants’ thoughts (Table 2). The sub‐sub‐themes specified these thoughts.

#### 3.1.1 The skills of the dog

A certified dog's task is to assist their owner in their everyday life and/or to warn against situations that could be unsafe. This was also reflected in the participants’ expectations (Table 2). They expected that the dog would assist them and thus they would achieve a less restrictive lifestyle.

That my dog will be good at bringing things, help me with my balance, be able to alert in different settings. That my dog will learn to be calm and focused on his work as we move out into society. (P3)

I get my freedom, I can get out whenever and wherever I want to, if I want to go out in the woods or go to Anytown [fictitious name given by authors]. That I will have good company every day. Someone who’s always there for me and helps me with things I find hard to accomplish on my own. (P100)

Participants with diabetes expressed their need for the dog to alarm for low and high blood sugar levels. In addition, they pointed out that the dog's ability to attract another person's attention in case of emergency was important.

That he [the dog] should be able to warn me when my blood sugar levels drop. Especially at night and during walks. Also at work. (D54)

The dog brings so much joy and company. I hope he [the dog] will be able to alert me when my blood sugar levels fall out of normal range. That he will be able to trigger my alarm if I go into diabetic coma at home. That he can get help if I end up helpless outside my home. (D32)

Several of the participants were afraid of having a diabetic coma. They hoped that the dog could decrease the risk of hyperglycemia as well as hypoglycemia. Skills of the dog that could decrease blood sugar fluctuations were closely related to the participants’ ability to feel secure.

#### 3.1.2 Security and independence

An increased sense of security permeated the participants’ expectations. In addition to security related to diabetes, they expected security in their everyday life. The participants also hoped that increased security would give them better confidence.

Feeling secure in everyday life. Feeling freedom in everyday life. Less need for help. Feeling needed, someone who needs me. (P73)

That it will give me a greater sense of security to go outside the home and to attend courses, meet friends, etc. (P10)

Expectations of increased security also made the participants reflect on their wish to become more independent. The term “independence” was frequently mentioned in several different contexts. Becoming more independent could mean both that the participants hoped to be able to expand their surroundings, but also that they would not have to rely on others as they were used to doing.

That he [the dog] will make me feel secure and free so I dare to go out on my own only accompanied by my dog. That I don´t have to wait for that one hour per week when the home‐help service comes for a walk. I want to be able to go out at the time I want, and when the weather is nice. (P100)

With help from a physical service dog I will become more independent and will have the chance to strengthen my self‐confidence and become more independent. I believe it will improve my living conditions, I will have a life that is simply better. (P35)

Several participants mentioned that one big relief would be that the dog would be able to pick up dropped items. The opportunity to become more independent seemed important for all participants, irrespective of the assistance dog type.

#### 3.1.3 The training

The participants also had expectations of the training. Some hoped that guidance from professional instructors would help them achieve the expectations they had of their future certified dog. The participants also expected that the training would mean growth and development for themselves.

I really look forward to starting the training, it will be good to have an instructor that can help with the training when we get stuck. I also hope to get input from other participants regarding what the dog can help with. My dog already knows some tasks but it´s always good to find new tasks to train in. I´m also looking forward to sharing experiences with other participants in the same or in similar situations. (P23)

That I will learn something new and develop. Improve the collaboration and communication between me and the dog. (P60)

The training was perceived important to ensure that the dog would be able to help them as expected.

#### 3.1.4 Overall life changes

The participants had an anticipation and desire to create a bond between themselves and the dog, and the overall expectations of their future assistance dog were high:

Improved quality of life, being more independent. Less stress, easier everyday life, better routines and improved health, both physically and mentally. My dog helps me to “calm down” in stressful situations which means that I can perform much better, utilise information and have less chaos in my brain. In turn, this means that I can cope more and that I can appreciate life, despite all the pain and physical deficiencies that I have. In addition, I can feel safe in situations I have problems to handle on my own, if I have my certified assistance dog with me. (P70)

That me and my family will feel safe. My mental health will improve. My chances to survive will increase. (D71)

The participants projected that receiving a certified dog in many ways would be life changing. Accordingly, the participants had high expectations of their future assistance dogs.

### 3.2 Perceived positive experiences

The perceived experiences in many ways reflected the expectations they had expressed before training their dog, see table 2. However, using a certified dog as an assistant had given them both positive as well as negative experiences.

#### 3.2.1 Health status

In general, the participants felt that their health status was better after having a certified dog by their side. Participants with epilepsy reported that their epileptic seizures had decreased due to the dog's ability to warn before emerging seizures, and participants with diabetes felt that the blood sugar levels had become more stable after the dog started to warn for fluctuations. More stable blood sugar levels had not only given the participants a greater feeling of security, it had also increased the security for relatives and friends.

She [the dog] prevents my seizures by warning me in time. (S29)

Buddy [fictitious dog name given by authors] helps me control my blood sugar levels. It makes it easier for me to keep my blood sugar within the “frames”. It is a huge benefit for my health and well‐being. I feel better when I´m with my dog and when I have my dog with me. Also, my attitude is better: It works! (D65)

That I can relax. My children (grown up) don´t have to worry. Liberating. (D48)

With assistance from the dog, the participants were able to reduce physical movements that had caused pain. As a result, the participants perceived that their physical functioning had improved. The presence of the dog also made it possible to remove focus from the pain. In addition, there were reduced levels of anxiety through the possibility to relax, better sleeping habits, less loneliness and an increased sense of happiness and freedom.

At the long‐term follow‐up the participants also experienced that the dog had positive effects on their pain levels. Reduced use of analgesics and other medications were also frequently mentioned.

I haven’t had the same need for strong painkillers (severe osteoarthritis in both hips) as during the years I had constant and debilitating pain. I have been able to stand still while the dog picks things up or closes the front door and I haven´t needed to get up to turn off the lamp, which hurts a lot, as well as to bend forward to take out the laundry. I haven’t needed that much medication for anxiety attacks either. (LPS00)

In addition, the participants mentioned, in accordance with the short‐term follow‐up, that the dog had positive effects on their mental health, blood sugar levels and epileptic seizures.

#### 3.2.2 Being active

The participants pointed out two different reasons for perceiving their life as more active at the short‐term follow‐up compared to baseline. The possibility to be more active due to the presence of the dog was one of them.

He [the dog] makes activities outdoors much easier, especially in the winter when he can help me get through the snow. (P60)

Another reason for being more active was the dog's need for outdoor activities and exercise. It was perceived positive since the outdoor activities increased their own well‐being and physique.

He [the dog] contributes to exercise and fresh air, which keeps me in good physical shape. It leads to improved well‐being and to being able to interact with other people and make new contacts. (H1)

Not everyone thought that the dog affected their amount of time spent on activities and stated that there was no difference from the time before having a certified dog. Although, at the long‐term follow‐up the majority still perceived that the dog increased their activity.

#### 3.2.3 Security and independence

The participants’ increased sense of security permeated their answers. The increased sense of security could be related to different aspects characterising the participants’ lives.

Max [fictitious dog name given by authors] makes my living so much safer with reference to my diabetes. He [the dog] warns me when I´m not able to feel that my blood sugar levels are low, for example at night, when I go to sleep, before I go out for a walk. At these times it could be life‐threatening for me if I didn’t adjust my blood sugar. (D54)

I feel calmer and safer when he´s [the dog] with me. (D79)

Closely related to the increased sense of security was the possibility to be more independent. The dog had expanded the participants’ surroundings and they also felt relieved to not constantly have to rely on others.

I can do things I couldn’t do before, for example to travel (on train), take walks in the woods etc. (D15)

I have a chronic disease so my dog can´t affect the disease itself but he increases my sense of independence. It also gives me improved ability to do things on my own with the certainty that for example a dropped phone or dropped car keys won’t be a problem = Security! (P17)

Independence was also frequently mentioned as a positive experience at the long‐term follow‐up. Participants at the long‐term follow‐up who expressed an increased feeling of independence all had a physical service dog.

#### 3.2.4 Social relationships

The participants also highlighted the positive effects that the dog had had on their social relationships. The dog had for instance meant that they had extended their network of contacts since they had come to know other dog owners. It had also expanded their opportunities to meet other people in general. At the long‐term follow‐up the majority experienced that the dog still had a positive effect on their social relationships.

Having a dog contributes to social interaction. Having a trained dog does even more. A lot of new contacts and many interesting conversations. I can have adventures that wouldn’t have been possible otherwise. For instance, I have lectures. There have simply been more social interactions. (D65)

He [the dog] is a friend‐maker. Who wants to talk to a middle‐aged woman in a wheelchair? But if she brings a big beautiful and clever dog, almost everyone wants to talk with her. (LP100)

Participants who did not have problems with social interactions before training a dog felt that the dog had had no impact on their social life at all.

He [the dog] does not affect my social situation. He is an ordinary dog and I’m an ordinary dog owner. (D16)

As at the short‐term follow‐up, one participant at the long‐term follow‐up pointed out that the reason for having an assistance dog was to receive assistive support, not to make new friends.

#### 3.2.5 The dog is the best thing that happened to me

The perceived positive experiences were directly related to their health state, but also connected to their emotional state of mind. Having a certified dog had for instance become life changing:

He [the dog] is the best thing that has happened to me. (P20)

The dog has given me a new life. (S52)

Having a physical service dog has meant a new life for me. Before I only thought of the practical things the dog could help me with. The practical things are important, but I had so much more, being outside strengthens the immune system, I became healthier, social contact is easier and I am never alone. (P23)

Several participants had high expectations of the dog and the dog's ability to help them. Additionally however, they experienced the dog's presence and help as even better than they could have imagined. The presence of the dog made the participants’ everyday life easier, happier and more meaningful.

#### 3.2.6 Additional positive experiences in the long‐term

In the long‐term the participants also perceived that the dog had become a great support in their everyday life and that it felt meaningful to have someone to look after and take care of.

He [the dog] is a “support”. (LP40)

Calmer, not that alone, better routines, someone to take care of, feel no mental obstacles. (LP19)

Additional positive experiences brought up at the long‐term follow‐up were the importance of the community with other dog owners that occurred during the training process, and the possibility to get help from competent instructors during the training, Table 2.

### 3.3 Perceived negative experiences

Beside the positive experiences, the dog also contributed to some negative experiences, Table 2. Several of these experiences were beyond the participants’ control and possibility to prevent.

#### 3.3.1 Negative attitudes and limited access to public places

Access to restaurants, grocery stores or other shops is not always permitted when bringing a dog. The participants stated that this occurred even though the dog wore the cape that identified the dog as a service or hearing dog carrying out a job. Not being allowed to bring the dog everywhere reduced their surroundings.

Since service/hearing dogs are so important for those of us with various disabilities it is necessary to have a more open society that allows us to bring the dog everywhere, for example to restaurants, into shops etc. (H1)

The only negative is the inaccessibility when I can´t bring him [the dog] with me. (P61)

The participants perceived the negative attitudes and their sometimes limited access to public places as offensive.

#### 3.3.2 Reduced freedom and feeling stressed

In certain situations, the participants expressed that the dog gave them reduced freedom and flexibility. For example, when undertaking travel for work purposes, when visiting friends who were allergic or in general when it felt difficult to bring the dog. Visiting friends with fur allergies was even sometimes impossible because of the dog.

Limited social situation. Can´t visit allergic friends etc. (D300)

In addition, the participants perceived it as stressful when not being able to give the dog enough exercise and activity.

#### 3.3.3 The training

The participants also identified a few obstacles with the training. For some of them the distance to the course of training was a problem, they had to travel a long way to be able to participate.

The training is challenging due to the long journey, for example 1200 kilometres round‐trip to take the certification test. (P3)

In addition, participants training their second dog felt that they had to go through steps in the training process with which they were already familiar. They wished that the course of training could have been more adapted to their previous knowledge.

This was the second physical service dog that I had trained so I had done a lot of the work before I started the course. The Swedish Association of Service Dogs needs to review the training for participants with their second or third dog, since everyone doesn´t need all days when conducting the suitability tests etc. I reckon that the government have paid a lot for my and Fia´s [fictitious dog name given by authors] training. (P33)

One participant also testified that the training was very time‐consuming. It had infringed upon the participant's job.

#### 3.3.4 Additional negative experiences in the long‐term

The ability to control the dog and the fact that the dog made the house dirty were additional negative experiences the participants brought up at the long‐term follow‐up (Table 2).

Big, happy dog that can be difficult to control in all situations. (LP19)

It´s dirtier at home, greater wear on furniture, clothes etc. (LP42)

Other negative experiences mentioned at the long‐term follow‐up were in line with the negative experiences mentioned at the first follow‐up.

## 4 DISCUSSION

In this article we have looked into the dog owners’ expectations before the training of the dogs, and their perceived experiences (both in short‐ and long‐term) after the dogs became certified. The expectations expressed before were closely related to the tasks the dog was intended to carry out after being certified. For instance, if the participant intended to train a diabetes alert dog, he or she expected the dog to be able to alarm for fluctuations in their blood sugar. Before training the dog, the participants also felt insecure both in their everyday life in general but also in specific situations, such as taking a walk or going to the grocery store. They hoped that the dog would contribute to an increased sense of security in these situations. The expectations were expressed irrespective of the assistance dog type they intended to train.

The expectations expressed before training a dog in many ways reflected the participants’ experiences after the dog was certified. The participants perceived that their desire for a safer and more independent everyday life had been fulfilled. In addition, the dog had affected the participants’ perceived health status. For instance, participants with a diabetes alert dog experienced that they were able to keep their blood sugar steady, and participants with a physical service dog thought that the dog had made them more mobile but also reduced their anxiety. In addition, the participants hoped that the dog would contribute to increased happiness. However, being accompanied by a certified dog turned out to be more overwhelming than they could have imagined in advance. They felt that the dog had given them a different life and become their best friend. In addition to the expectations, other positive experiences were identified. For instance, through the dog, the owner was invited to a more physically active lifestyle and the dog was also a catalyst for social interactions. The dog being a catalyst for social interactions has also been established in previous research (Camp, 2001; Hubert, Tousignant, Routhier, Corriveau, & Champagne, 2013). The participants also stated that the dog reduced family members worrying. This is consistent with results presented in a recent study (Bibbo, Rodriguez, & O'Haire, 2019).

The positive experiences brought up directly after the dog had become certified also reflected the positive experiences identified over time. Worth noting though was that the bond between the dog and the owner seemed to become stronger and stronger. Furthermore, when entering the project none of the participants thought that the dog would mean something negative. However, negative experiences were brought up and these experiences remained. For example, restrictions on bringing the dog into public places and negative attitudes from other people. The findings in our study are consistent with what has been found in previous studies (Camp, 2001; Herlache‐Pretzer et al., 2017; Rodriguez et al., 2019). Rodriguez et al. analysed data from a cross‐sectional open‐ended survey. They identified that service dog owners perceived physical and psychosocial benefits but also drawbacks such as public access, lifestyle adjustments and dog behaviour (Rodriguez et al., 2019).

In a previous study, potential consequences for the participant's HRQoL, well‐being and activity level, of having a certified service or hearing dog, were studied with generic instruments (Lundqvist et al., 2018). The results indicated that there were positive effects of having a certified dog. This study can be seen as a complement to the previous study. It demonstrates the importance of supplementing quantitative data with qualitative data to identify and depict the multifaceted effects of an intervention. Compared to the previous study, where the results were statistically uncertain, the results from this study are convincing. The participants perceived that the certified dog had a positive impact on both their physical and mental well‐being.

When summarising existing research findings, indications of positive consequences of having a certified dog in terms of HRQoL, well‐being and activity level, can be seen (Lundqvist et al., 2018). In addition, economic analyses show that certified service dogs are cost saving in comparison to regular companion dogs (Lundqvist, Alwin, & Levin, 2019) and, as has been shown in this study, participants perceive their certified dog as an assistive support that is worth its weight in gold. Is this research providing enough evidence to establish guidelines regarding prescription and subvention of an assistance dog as a supportive aid in Sweden? Only time will tell.

The limitations of this study should be acknowledged. First, since the participants were self‐selected there may be a risk of self‐selection bias. The selection‐bias may lead to the sample not being representative, or the opposite, the sample may feel committed to take part and thereby provide more meaningful insights than if being randomly selected. We believe that the selection‐bias in this case is minimal if generalising our results to people training a certified dog on their own. However, generalising the results to those purchasing a fully trained dog may not be possible. Secondly, the design of the questions (open‐ended) may have influenced the responses we received. However, the participants had the opportunity to add other aspects, in regard to having a certified dog, than those highlighted in the questions. Despite these limitations, our study gives valuable insights into what service and hearing dogs can contribute, and how that affects their owners. Hence, this study offers valuable input to decision‐makers when prioritising individually adapted care to people who can potentially benefit from being assisted by a certified dog.

## 5 CONCLUSIONS

This study shows that individuals who are supported by an assistance dog experience the dog as an invaluable help in their everyday life. They feel that the dog has a positive impact on their health status. In addition, they perceive that the dog gives them the ability to become more independent and active and feel more secure. The dog also strengthens their social relationships. The poor understanding in society of the role played by the assistance dog is one negative part of assistance dog ownership. By improving the owners’ lives in many ways, a certified service or hearing dog is a novel and important assistive support for people with a functional or health impairment.

## Additional file 1

### Questions asked at baseline:

1. What are your expectations of training a certified service or hearing dog?
2. How do you think the dog will influence your situation after becoming certified?

## Additional file 2

### Questions asked at first and second follow‐up:

1. What does your certified dog mean for your social situation?
2. What does your certified dog mean for your well‐being?
3. How does your certified dog affect your ability to perform activities?
4. Has the dog had any significant influence on your medical situation and your perceived illness?
5. Has the dog given you negative experiences?
6. Is there anything else you want to add?

## CONFLICT OF INTEREST

There were no conflicts of interest.
